# Differentiation between first and second messenger effects of cGMP

**DOI:** 10.1186/2050-6511-16-S1-A84

**Published:** 2015-09-02

**Authors:** Erich Schneider, Sabine Wolter, Fanni Dittmar, Gonzalo Fernández, Roland Seifert

**Affiliations:** 1 Institute of Pharmacology, Hannover Medical School (MHH), Germany; 2 Current address: Dept. of Medical Biochemistry and Biophysics, KaroIinska Institute, Sweden

## Background

The secondary messenger cGMP does not only play an important role in the cardiovascular system (vasodilation and inhibition of platelet aggregation), but also regulates the functions of numerous cell types like immune cells, adipocytes or neurons [1,2]. Besides its inactivation by phosphodiesterases, cGMP is exported from cells by the organic anion transporter 2 (OAT2) [3] or by multi-drug-resistance proteins (MRP) [4]. Thus, cGMP may also cause effects from the extracellular side by acting as a first messenger on cell surface receptors. In fact, extracellular effects of cGMP have been described in the past. A selection of papers is listed in Table 1. A more detailed review is provided in [1].

**Table 1:** Examples for extracellular first messenger effects of cGMP:

Model	extracellular cGMP effect	Ref.
rat model of colorectal pain	analgesic (oral cGMP)	[7]

oxidative glutamate toxicity in HT22 cells	neuroprotective	[8]

kainate receptor-mediated neurotransmitter release	inhibition	[9]

glycine receptor-induced chloride current	accelerates desensitization	[10]

Additionally, extracellular cGMP could be degraded by ecto-phosphodiesterases and the degradation products (GMP and guanosine) may have first messenger functions of their own. In summary, the biological functions of cGMP are much more versatile than previously acknowledged. Therefore, our project aims at differentiating between first- and second messenger functions of cGMP and the other cyclic nucleotides (cNMPs) cAMP, cUMP and cCMP in various cellular systems and functional readouts.

## Methods

The second messenger functions of cGMP were imitated by the membrane-permeable acetoxymethyl ester analogue (cGMP-AM), which releases cGMP after intracellular hydrolysis. Unmodified cGMP was applied for the characterization of extracellular first messenger effects. In a similar way, the first- and second messenger effects of cAMP, cCMP and cUMP were characterized. As cellular models and functional readouts we used (1) flow cytometric determination of apoptosis (annexin V/propidium iodide staining) of wildtype S49 lymphoma cells (S49 wt) and their kinase-negative counterparts (S49 kin^-^), (2) flow cytometric cell cycle determination of HEL (human erythroleukemia) cells, (3) ELISA-based quantitation of T-cell receptor-mediated (anti-CD3 antibody stimulation) IL-2 production of HuT-78 lymphoma cells and (4) determination of chloride flux in Calu 3 lung epithelial cells with the chloride-sensitive dye MQAE (N-(ethoxycarbonylmethyl)-6-methoxyquinolinium bromide).

## Results

None of the unmodified cyclic nucleotides (cAMP, cGMP, cUMP, cCMP) caused apoptosis in S49 wt or S49 kin^-^ cells, and the cell-permeable AMs of cAMP, cGMP and cUMP were not active either. Interestingly, guanosine, a degradation product of cGMP, induced apoptosis. Moreover, cCMP-AM exerted a surprising pro-apoptotic effect on both S49 wt and S49 kin^-^ cells. Cell cycle analysis of HEL cells revealed that cGMP-AM increased the percentage of cells in the SubG1 phase, but reduced the proportion of cells in the G2/M phase.

The T cell receptor-mediated IL-2 production of HuT-78 cells was inhibited by cGMP, but not by cGMP-AM. The CFTR-mediated chloride flux in Calu 3 cells was slightly stimulated by cAMP-, cGMP- and cUMP-AM, but cCMP-AM had no significant effect. No significant stimulation of chloride flux in Calu 3 cells was observed with cell-impermeable unmodified cNMPs.

## Conclusions and outlook

Our data suggest that first and second messenger functions of cGMP depend on cell type and functional readout. Thus we plan to perform further studies with a variety of model systems in order to generate a “functional profile” of first- and second messenger effects of cGMP. Moreover, the surprisingly strong pro-apoptotic effect of cCMP-AM on S49 cells warrants similar studies with cyclic pyrimidine nucleotides. cCMP and cUMP now fulfil most of the criteria for second messenger functions [5]. Finally, it was recently reported that extracellular cIMP causes vasoconstriction in porcine coronary arteries [6]. Thus, cIMP should also be included in the list of potential first/second messengers and characterized more closely in future studies.
